# Metatranscriptomic responses and microbial degradation of background polycyclic aromatic hydrocarbons in the coastal Mediterranean and Antarctica

**DOI:** 10.1007/s11356-023-30650-1

**Published:** 2023-11-07

**Authors:** Alicia Martinez-Varela, Gemma Casas, Naiara Berrojalbiz, Daniel Lundin, Benjamin Piña, Jordi Dachs, Maria Vila-Costa

**Affiliations:** 1 Department of Environmental Chemistry, Institute of Environmental Assessment and Water Research, IDAEA-CSIC, c/ Jordi Girona 18-26, 08034 Barcelona, Catalunya Spain; 2 Centre for Ecology and Evolution in Microbial Model Systems, EEMiS, Linnaeus University, 35195 Kalmar, Sweden

**Keywords:** Polycyclic aromatic hydrocarbons (PAH), Metatranscriptomics, 16S amplicon sequencing, PAH biodegradation rates, Antarctica, Mediterranean

## Abstract

Although microbial degradation is a key sink of polycyclic aromatic hydrocarbons (PAH) in surface seawaters, there is a dearth of field-based evidences of regional divergences in biodegradation and the effects of PAHs on site-specific microbial communities. We compared the magnitude of PAH degradation and its impacts in short-term incubations of coastal Mediterranean and the Maritime Antarctica microbiomes with environmentally relevant concentrations of PAHs. Mediterranean bacteria readily degraded the less hydrophobic PAHs, with rates averaging 4.72 ± 0.5 ng L h^−1^. Metatranscriptomic responses showed significant enrichments of genes associated to horizontal gene transfer, stress response, and PAH degradation, mainly harbored by Alphaproteobacteria. Community composition changed and increased relative abundances of Bacteroidota and Flavobacteriales. In Antarctic waters, there was no degradation of PAH, and minimal metatranscriptome responses were observed. These results provide evidence for factors such as geographic region, community composition, and pre-exposure history to predict PAH biodegradation in seawater.

**Supplementary Information:**

The online version contains supplementary material available at 10.1007/s11356-023-30650-1.

## Introduction

Large quantities of polycyclic aromatic hydrocarbons (PAH) and other semivolatile aromatic-like compounds (SALCs) are released to the environment and transported globally by long-range atmospheric transport and deposition (González-Gaya et al. 2016). The dissolved complex mixture of PAH and SALCs enters the oceanic water column mainly by atmospheric air-water diffusive exchange, with an estimated global input of 400 Tg year^−1^ (González-Gaya et al. 2016, 2019a). Once in the ocean, they represent a major fraction of the anthropogenic dissolved organic carbon (ADOC) (Vila-Costa et al. 2020; Trilla-Prieto et al. 2021). While PAHs occur in relatively low abundances in the marine water column, their potential bioconcentration, toxicity, recalcitrance, co-occurrence, and the possibility of generation of secondary reactive metabolites pose a threat to ecosystems (Hylland 2006). Surprisingly, the biological pump sinking fluxes of PAH and SALCs are estimated to represent only 1% of the total input to the surface ocean, suggesting the largest share is transformed in the upper ocean, mainly by biodegradation (Ghosal et al. 2016; González-Gaya et al. 2019a). Unfortunately, the scarcity of experimental biodegradation field studies under environmentally relevant concentrations hampers the assessment of the magnitude of this sink and how this process impacts microbial communities. Microbial degradation of PAHs, and effects of PAHs on microbial communities, has mainly being addressed under scenarios of oil spills, but to a lesser extent for coastal environments, also receiving important inputs of PAHs from land or from atmospheric deposition (Berrojalbiz et al. 2011; Casal et al. 2018).

The metabolic pathways for the microbial transformation of PAH under aerobic conditions, from primary biodegradation to their total breakdown to CO_2_, are well known for isolated bacteria and typically start with the hydroxylation of an aromatic ring via a ring hydroxylating dioxygenase (RHD) (Mallick et al. 2011; Ghosal et al. 2016). Many bacterial strains capable of metabolizing these compounds have been identified and successfully isolated from oil spills (Mittal and Singh 2009; Gutierrez et al. 2013; Lamendella et al. 2014; Mason et al. 2014). These so-called hydrocarbonclastic bacteria (HCB), including facultative and obligate hydrocarbon degraders, harbor a large arsenal of genes related to PAH catabolism and assimilation, sensing hydrocarbon presence, positive PAH chemotaxis, PAH-specific detoxifying mechanisms, facilitators of gene transfer of PAH catabolic genes between the microbial community, or the production of biosurfactants to increase hydrocarbons bioavailability (Joye et al. 2016; Top and Springael 2003). Genomics and transcriptomics, and their environmental counterpart, metagenomics and metatranscriptomics, have revealed some of the multiple strategies employed to cope against PAH toxicity and the metabolisms used for PAH biodegradation under lab and field conditions (Rivers et al. 2013; Cerro-Gálvez et al. 2019; Cerro-Gálvez et al. 2021). The degree of biodegradation of PAH is influenced not only by the composition of the naturally occurring microbial community and factors driving the microbial consortia functioning, but also by the environmental factors directly influencing kinetics such as temperature, PAH concentrations, and cell-water partitioning. Nutrient limitation and the level of pre-exposure and acclimation to ADOC pollution levels have been identified as relevant factors affecting the pollutant-bacteria interactions (Hudak and Fuhrman 1988; Cerro-Gálvez et al. 2021). It has been suggested that environmental exposure to ADOC may trigger the horizontal dissemination within the community of the genes required to cope with, or metabolize ADOC, via mobile genetic elements (MGE) by horizontal gene transfer (HGT) and other mechanisms, as it has been observed after antibiotics exposures in riverine bacterial communities (Top and Springael 2003; Gillings 2013; Lekunberri et al. 2018). Unfortunately, most previous studies on bacteria exposure to PAHs focused on the microbial responses after accidental oil discharges, related to high concentrations of PAH, and punctual occurrence (Head et al. 2006; Gutierrez et al. 2013; Gallego et al. 2014; Crisafi et al. 2016; Dombrowski et al. 2016; Atashgahi et al. 2018). The microbial responses to the ubiquitous, chronical, and background concentrations of PAH pollution found in the oceans have been much less studied, and this dearth of data hampers the correct assessment of biodegradation, as well as its inclusion into biogeochemical models.

The marine environment is key to determine the variability of PAH biodegradation and PAH influences on microbial communities since it offers different contrasting environments in terms of pollution, nutrient limitations, and temperatures. For instance, the semi-enclosed Mediterranean basin contains temperate waters encircled by highly populated areas, being the Gibraltar strait the only entrance of seawater from the Atlantic Ocean. Thus, riverine run-off and atmospheric inputs are the most relevant PAH inputs (Lipiatou et al. 1997; Berrojalbiz et al. 2011; Castro-Jiménez et al. 2012). Oppositely, the Southern Ocean is a polar environment connected to all major oceanic basins and far from any major pollution emission source. Under these conditions, diffusive air-sea exchange and snow and glacier melting represent the main PAH entry to the polar water column (Cabrerizo et al. 2014; Casal et al. 2019). While the genes responsible for PAH degradation are ubiquitous in the surface ocean (González-Gaya et al. 2019b; Trilla-Prieto et al. 2021), the kinetics and degradation potential for the different marine regions remain unknown.

The goals of this study are to quantify PAH biodegradation rates and to describe microbial responses to the exposure to environmentally relevant concentrations of PAHs in two contrasting marine environments by means of biogeochemical measurements, changes in community composition using 16S amplicon sequencing, and changes in gene expression profiles by metatranscriptomics. The two contrasting sites are the Mediterranean Sea, representing a polluted site with a pre-adapted microbiome expectedly harboring high gene plasticity, and the Maritime Antarctica, representing a remote site receiving pollution from different sources (mainly wet deposition), with a lower temperature, and probably with a lower microbial pre-adaptation traits towards ADOC pollution.

## Material and methods

### Description of the experiments

The same experiment was carried out at two different locations, Blanes Bay Microbial Observatory (BBMO), North-Western Mediterranean Sea (41° 40′ 13″ N, 02° 48′ 01″ E, August 8, 2017), and South Bay at Livingston Island (South Shetlands Islands, Antarctica, 62° 39′ 26.3″ S 60° 23′ 18.4″ W, February 27, 2018). The experiment consisted of a 48-h incubation of surface seawater spiked with environmentally relevant concentrations of individual PAHs (Table S1). The PAH spike was achieved using PAH-Mix 9 from Dr Ehrenstorfer GmbH in cyclohexane. Prior to the addition of PAHs, 2-L glass bottles were pre-cleaned and baked at 400 °C. The PAH mix was added to the treatment bottles, while the control bottles received the same volume of only the solvent (cyclohexane) without a PAH spike. This solvent was allowed to evaporate for 3 h before seawater was added to prevent any potential microbial response due to the presence of the solvent. Duplicates were done for each experimental condition. At each experiment location, surface seawater (from 5-m depth) was collected using 20-L metal carboys. The 48-h incubations took place in the dark and at in situ monitored constant temperature, specifically, 2 and 20 °C for the Antarctic and Mediterranean experiments, respectively. Additionally, abiotic controls were run using HPLC-grade water with two replicates and were incubated and proceeded as the live treatments.

### PAH analyses

Briefly, 1 L of seawater was filtered through a pre-combusted glass fiber filter (GF/F, 47 mm, Whatman) to collect the particulate-phase PAHs. Filters were stored wrapped in aluminum foil and in a zip bag at −20 °C until their extraction. Once in the laboratory, each filter was freeze-dried, weighed, and spiked with 50 ng of a mix containing five recovery standards (Naphthalene-d8, Acenaphthene-d10, Phenanthrene-d10, Chrysene-d12, and perylene-d12; Sigma-Aldrich). PAHs were extracted in a clean lab by sonication with a mixture of hexane-acetone (1:1 v/v) solution, a process that was repeated three times. The supernatant containing PAHs was collected in a pre-combusted pear-shaped glass flask, concentrated using a rotary evaporator and solvent exchanged to hexane. These extracts were then purified using a glass column filled with 100 mg of anhydrous sodium sulfate over 1 g of neutral alumina (aluminum oxide 90, activated at 450 °C for 12 h) and eluted with 15 ml of hexane/dichloromethane (3:1, v/v). The extracts were further concentrated under a gentle stream of purified N_2_ to a final volume of 100 μL and transferred to an injection amber glass vial.

The sampling of dissolved PAHs was performed in situ on the field immediately after filtration. Dissolved-phase PAHs were collected using C18 solid-phase extraction cartridges (Agilent, 6 ml, 500 mg, 40 μ, 30/pk) using a vacuum pumping system. These cartridges were pre-conditioned with 6 ml of hexane, 6 ml of propanol, and 6 ml of HPLC-grade water with 2% of propanol. Each cartridge was spiked with 50 ng of the same recovery standard mix used for the particle phase. After extraction, the cartridges were kept under vacuum for 20 min to remove any residual water and stored at −20 °C until further processing in a clean lab. PAHs were eluted from the cartridges with 12 ml of a mixture of hexane:dichloromethane (1:1, v/v). The extracts were solvent exchanged to isooctane and concentrated under a gentle stream of purified N_2_ to a final volume of 100 μL.

PAHs analysis was conducted by gas chromatography coupled with a mass spectrometer (GC-MS, Agilent 5975C) based on an established method with minor modifications (Casas et al. 2021). The chromatographic separation for PAHs was carried out using an Agilent DB-5MS column (30 m, 0.25 mm internal diameter, 0.25 μm film thickness). Two microliters of sample were injected in split less mode. The separation was achieved with a 30 m × 0.25 mm i.d. × 0.25 μm DB5 capillary column (Agilent). The acquisition was performed in selected ion monitoring (SIM). A total of 50 ng of three internal standards were spiked into the final vial for quantification (Anthracene-d10, Pyrene-d10, Benzo(b)fluoranthene-d12) before the instrumental analysis. The analytical procedure for dissolved and particle-phase PAHs was validated by determining the recoveries of the surrogates for each sample. Analytical blanks were analyzed following the same procedure than field samples. The following 13 PAHs were quantified: Fluorene, Phenanthrene, Anthracene, Pyrene, Fluoranthene, Crysene, Benzo(a)anthracene, Benzo(b)fluoranthene, Benzo(k)fluoranthene, Benzo(a)pyrene, Dibenzo(a,h)anthracene, Indeno(1,2,3-cd)pyrene, and Benzo(ghi)perylene. Dissolved and particle phase PAH concentrations were determined at initial time and after 48 h from the start of the incubation. PAH biodegradation rates were estimated as a mass balance between concentrations at T48 versus T0 (see text S1 in Supplemental Material).

### Nucleic acid extraction and sequencing

Samples for metatranscriptomics (mRNA) and 16S rDNA libraries (DNA) were collected after 3 h and 48 h of incubation, respectively, to capture gene expression profiles and changes in community composition after PAH additions. Seawater samples were pre-filtered through a 20-μm nylon mesh, then pre-filtered through a 3 μm pore-size 47-mm polycarbonate filters, and then filtered onto 0.2 μm pore-size 47-mm polytetrafluoroethylene filters, in order to capture the free-living bacterial cells fraction. For metatranscriptomic analyses, 1.2 L of seawater from each bottle were filtered, and the filtration step lasted no longer than 15 min to minimize RNA degradation during handling. Filters were placed into 1 mL of RNAlater (Sigma-Aldrich, Saint Louis, MO). Total RNA was extracted, DNA removed, and mRNA amplified as previously described (Poretsky et al. 2009), with the only modification that total RNA was extracted with mirVana isolation kit (Ambion), after removing the storage reagent by centrifugation. Amplified mRNA was sequenced at the National Center for Genomic Analysis (CNAG, Barcelona, Spain) using Illumina high output model HS200 2 × 100 bp v4.

For community composition analyses, up to 0.4 L of seawater was filtered. Each filter was placed in 1 mL lysis buffer (50 mM Tris HCl, 40 mM EDTA, 0.75 M sucrose). All filters were stored at −20 °C. DNA extraction for 16S rDNA amplicon sequencing was performed as described in Vila-Costa et al. 2018. Ampification of 16S rRNA gene fragment was performed using  primers 515F-Y (5′- GTGYCAGCMGCCGCGGTAA) and 926R (5′-CCGYCAATTYMTTTRAGTTT) (Parada et al. 2016). The PCR consisted of an initial denaturation step at 95C for 5 min and 35 cycles at 95C (30 s), 58C (30 s), and 72C (40 s), followed by a final elongation step at 72C for 10 min. Sequencing was performed in an Illumina MiSeq sequencer (2 × 250 bp, Research and Testing Laboratory; http://rtlgenomics.com/).

The complete nucleotide sequence datasets generated in this study have been deposited in the European Nucleotide Archive (ENA) at EMBL-EBI under accession number PRJEB55141.

### Prokaryotic cell abundance

The abundance of prokaryotic cells was estimated by flow cytometry as described elsewhere (Falcioni et al. 2008). Briefly, heterotrophic non-pigmented total bacteria enumeration was performed by staining 0.4 mL of seawater with 4 μL of a 10 SG1 (Molecular Probes) solution for 10 min. These stained samples were run through a FACSCalibur flow cytometer. Bacteria were counted with CellQuest and PaintAGate software (Becton Dickinson, Palo Alto, CA) from the side scatter versus green fluorescence plot.

### Nutrient analysis

Nitrate(NO_3_^−^), nitrite (NO_2_^−^), ammonium (NH_4_^+^), and phosphate (PO_4_^3−^) were determined in 15 mL of seawater kept at −20 °C until analysis in the laboratory. The dissolved nutrients were quantified by standard segmented flow with colorimetric detection (Grasshoff et al. 1999) using a SEAL Analyzer AA3 HR. The limits of detection were 0.006 μM for NO_3_^−^, 0.003 μM for NO_2_^−^, 0.003 μM for NH_4_^+^, and 0.01 μM for PO_4_^3−^, as defined as three times the standard deviation at 50% diluted samples (ten replicates).

### Bioinformatics

For 16S amplicon reads, spurious sequences and primers were trimmed using Cutadapt v.1.16 (Martin 2011), using a cut-off minimum phred quality score of 20 (1% error rate). DADA2 v1.4 was used to differentiate the 16S V4-5 amplicon sequence variants (ASVs) and remove chimeras (parameters: maxN = 0, maxEE = 2,4, trunclen = 220,180; Callahan et al. 2016). DADA2 allows to infer exact variants up to 1 nucleotide difference, being threshold-free, by using the quality score distribution in a probability model. Taxonomic assignment of the ASVs was performed with the SILVA algorithm classifier against SILVA database release 138 (Quast et al. 2013). The dataset accounted for a total of 2326 unique ASV and the minimum sequencing depth was 24,550. Rarefraction to the minimum sequencing depths was done with rrarefy() function from vegan v1.4-4 package in R (Oksanen 2015). ASVs classified as mitochondria or chloroplast were removed. Archaea accounted for <1% of the total pool of reads and were discarded from the analyses.

For the metatranscriptomes, cDNA sequences were quality trimmed with Cutadapt (Martin 2011) and Sickle (https://github.com/najoshi/sickle). The remaining rRNA and tRNA sequences were quantified and removed using the Bowtie2 mapping program (Langmead and Salzberg 2012). Subsequently, high-quality reads were de novo assembled using the MEGAHIT program (v1.1.2) (Li et al. 2016) with default parameters. Open reading frame (ORF) prediction was done using Prodigal (Hyatt et al. 2010). Contigs were then aligned to the NCBI RefSeq database using the Diamond aligner, v0.9.24, (Buchfink et al. 2015) in blastx mode with default parameters. Functional SEED classification and taxonomic affiliation were assigned with MEGAN 6.7.3 (Huson et al. 2016). Transcripts were mapped to the open reading frames (ORFs) using bowtie2 (v2.3.5.1) and quantified using Samtools (v1.9). Further analysis was done in the R/tidyverse environment.

Search for specific PAH degrading and transposases genes within the metatranscriptomes was performed using Hmmersearch with default parameters against the specific Pfam hmmer profiles. The list of the specific Pfam profiles used is given in Table S2 and was downloaded from “Pfam” database (Mistry et al. 2021).

### Identification of hydrocarbonoclastic bacterial genera

The detection of specific HCB genera was performed following Martinez-Varela et al. (2021, 2022). Briefly, a list of hydrocarbonoclastic bacterial (HCB) genera was compiled from the literature (Lozada et al. 2014; Karthikeyan et al. 2020), comprising genera either identified in hydrocarbon polluted environments by oil spills, either observed to have stimulated growth following exposure to hydrocarbons, or harboring hydrocarbon catabolic activity. These were either from isolates or from marine environments after metagenome-assembled genomes (MAGs) reconstruction. ASV were filtered at the genus level, including ASVs and transcripts with taxonomical affiliation matching to those targeted HCB groups. All HCB-ASV and transcripts within the same genus were assumed to share similar metabolism. Additionally, a subset of ASV assigned to HCB genera were compared with BLAST against a relevant isolate from the same genus known to degrade PAH or retrieved from oil spill-impacted sites. Those ASVs with >97% of similarity were considered as true match.

### Statistical analyses

Shannon diversity indexes (H′) of 16S amplicon libraries were calculated with diversity() function from the “vegan v1.4-4” package. Differences in community composition between treatments or sites were elucidated with a permutation analysis of variance (PERMANOVA, function Adonisin “vegan v1.4-4” package in R). The function foldchange from “gtools” package (Wickham et al. 2019) was used to determine fold changes between treatments (PAH vs control). *T*-tests between treatments and controls were done using the “t.test” function from R package “stats”, with a *p*<0.05 significance threshold. Further graphs were plotted using the “ggplot2” package, also in R environment (Wickham 2009).

## Results and discussion

Microbial communities from coastal seawater in NW Mediterranean and Antarctic Peninsula were exposed for 48 h to environmentally relevant concentrations of a mixture of PAH. Differences in PAH turnover rates, gene expression profiles, and changes in community structure were analyzed to understand responses under biogeochemical realistic conditions in two contrasted environments.

### Environmental settings

South Bay was characterized by cold (1.5 °C) and nutrient-rich waters during the Antarctic austral summer, hosting an initial bacterial community dominated by Bacteroidota (54.3 ± 1.1%) and Proteobacteria, mostly Gammaproteobacteria (26.6 ± 0.1 %) and a minor contribution of Alphaproteobacteria (Table S3, Table S4, and Fig. S1). In contrast, a warm (26.4 °C) and oligotrophic system characterized the Mediterranean coastal sampling site. This site was dominated by the SAR11 clade and other Alphaproteobacteria (49.28 ± 0.5%) (Table S4 and Fig. S1). Diversity indexes based on 16S rDNA amplicon sequencing showed that the Mediterranean microbiome was significantly more diverse than the Antarctic microbiome (H′ 4.61 ± 0.10 and 1.56 ± 0.30 respectively, Table S3). Abundances of heterotrophic cells quantified by flow cytometry were 1.8-fold to 4.4-fold higher in the Mediterranean compared to Antarctica (Table S3). The Antarctic heterotrophic bacterial community was dominated by high DNA (HNA) cells, whereas in the Mediterranean, the bacterial community was evenly distributed between HNA and low DNA (LNA) cells.

Dissolved and particle phase concentrations of PAHs were in the low ng L^−1^ range at both sites (field in situ concentrations correspond to the concentrations in controls in Table S1). For example, concentrations of phenanthrene were 2.2 ng L^−1^ and 3.1 ng L^−1^ at the Antarctic and Mediterranean coastal sites, respectively. A larger number of individual PAHs could be detected in Antarctica, especially those with five aromatic rings. This is probably due to the role of snow-melting flushing of soil-bound PAHs and snow inputs of PAHs in coastal Antarctica (Casal et al. 2018). Conversely, in the Mediterranean, most PAHs enter the marine environment by diffusive air-water exchange, which is mainly for PAHs with two to four aromatic rings (Castro-Jiménez et al. 2012). The profile of PAHs in both the Mediterranean and Antarctica was dominated by phenanthrene, and other PAHs with two–four aromatic rings such as Fluorene, Pyrene, and Fluoranthene. These distributions and levels are consistent with those reported previously in South Bay (Casal et al. 2018; Cerro-Gálvez et al. 2019), and in the Catalan Sea (Dachs et al. 1997; Guitart et al. 2004; Berrojalbiz et al. 2011), but in the lower range of PAHs concentrations reported for other coastal Mediterranean regions (Table S5).

The concentrations of organic pollutants in the water column are the result of the interplay of the magnitude of sources as well as the biogeochemical cycle and sinks in the water column. The Mediterranean Sea is surrounded by densely populated regions and thus inputs to seawater are higher than in the Southern Ocean (Castro-Jiménez et al. 2012; Casal et al. 2018). Biodegradation is a key sink of PAHs, accounting for the removal of 99% of PAH inputs in the marine environment (González-Gaya et al. 2019b). Thus, differences in the degree of microbial degradation rates could explain the fact that even though PAH inputs are higher in the Mediterranean than in Antarctica, their concentrations in seawater are similar at both sites. The plausibility of this scenario is reinforced by the evaluation of microbial degradation reported here.

### PAH biodegradation

**Fig. 1:**
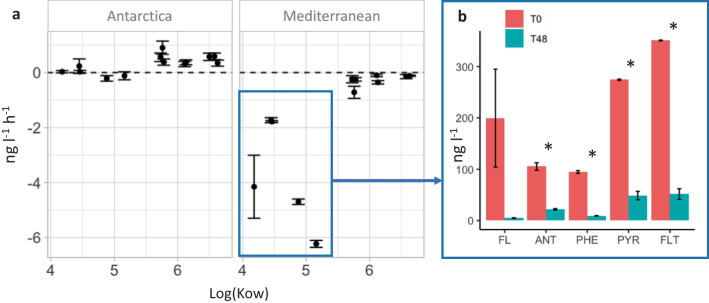
**a** PAH removal rates for the PAH amended samples in the Antarctica and the Mediterranean, calculated as the difference of concentrations between time 0 and 48 hours (units in ng L^−1^ h^−1^). **b** Concentrations of the PAH compounds with fastest removal rates in the Mediterranean (units in ng L^−1^). Significant differences between initial and final exposure time are indicated with asterisk (*), based on *t*-test (*p*-value < 0.01). (FL Fluorene, ANT Anthracene, PHE Phenantrene, PYR Pyrene, FLT Fluoranthene, Kow Octanol—water constant)

Dissolved and particle phase PAH concentrations were quantified at time 0 and after 48 h of PAH exposure (Fig. 1). In both Mediterranean and Antarctic sites, 96–97% of LMW PAHs were found in the dissolved phase, whereas HMW PAHs showed an even distribution between dissolved and particle phases in Antarctica (47% particulate, 53% dissolved) and mostly found in the dissolved phase in the Mediterranean (85% on average). This difference between the fraction in particle-phase is consistent with Mediterranean water being oligotrophic, while South Bay in the Southern Ocean is considered mesotrophic. The comparison of final and initial concentrations allows to estimate the percentage of removal of PAH within a 48-h period. No significant PAH degradation was observed in the Antarctica experiments. Conversely, in the Mediterranean, a 29.9 ± 1.3% decrease in the concentration of the total 13 PAH (∑_13_PAH) from seawater after 48 h of incubation was observed. This decrease was due to the fast degradation of the less hydrophobic PAHs (Anthracene, Phenanthrene, Pyrene, and Fluoranthene, log Kow < 5.5) (Fig. 1), which are mainly found in the dissolved phase. In fact, the less hydrophobic individual PAH removal rates in the Mediterranean, calculated as the difference of the total PAH concentrations between time 0 and 48 h, ranged from 1.7 ± 0.2 to 6.2 ± 0.1 ng h^−1^, averaging 4.72 ± 0.5 ng h^−1.^. The concentrations of the heavier, more hydrophobic PAHs (five aromatic rings or more) showed no significant changes during the 48-h period. We consider that the observed removal rates reflect biodegradation, since experiments were run in the dark and, therefore, no direct photo-degradation could occur. Abiotic controls did not show such a decrease of low MW PAHs, as shown in previous PAH degradation experiments (Martinez-Varela et al. 2022).

Numerous abiotic and biotic factors can influence the extent of biodegradation (Ghosal et al. 2016). For example, PAH solubility and bioavailability increase with decreasing log Kow, with a larger fraction of PAHs in the dissolved phase. Therefore, PAHs with low log Kow tend to be removed faster by microorganisms than more hydrophobic ones (Froehner et al. 2012), which is consistent with our observations in the Mediterranean. In addition, higher temperatures decrease the partitioning of PAHs to particles, thus with a higher fraction of PAHs in the dissolved phase as observed at the Mediterranean site in comparison with the Antarctic site. Thus, at higher temperatures, a larger fraction of PAH molecules are bioavailable for microorganism uptake and metabolism. The degradation metabolism will also be faster, in principle at higher temperatures, as the temperature differences between the 2 °C in Antarctica and 20 °C in the Mediterranean were notable. Although our results show no significant removal of PAH during the experiment carried out in the maritime Antarctica, biodegradation of PAH and other petroleum hydrocarbons by marine microorganisms has been reported in a number of studies in similarly cold regions (Yakimov et al. 2004; Brakstad and Bonaunet 2006; Crisafi et al. 2016; Garneau et al. 2016), although with longer exposure times (15 – 30 days). These results suggest that the Antarctica’s system responded to PAH presence much slower than the Mediterranean system, in which 48 h of incubation were sufficient for the naturally occurring microbial community to remove a large fraction of low MW PAHs.

This work distinguishes itself from previous reports on PAH degradation by conducting experiments at environmentally relevant concentrations, which are lower than those typically encountered in accidental oil spills.

### Metatranscriptomic responses to PAH exposure

**Fig. 2:**
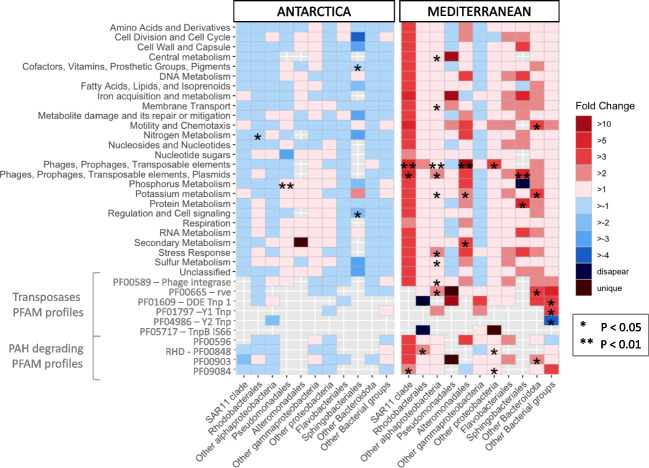
Fold changes in the contribution of functional categories for different taxonomic affiliations to the metatranscriptomic pool in treatments versus controls after 3 h of PAH exposure (the asterisk (*) symbols indicate significant changes between control and PAH exposed communities based on *t*-test at *p*>0.05 and the asterisk symbols (**) at *p* > 0.01). Annotation of significantly different expressed genes in SEED, egg-nog, KEEG, and COG can be found in Table S8

Both communities were exposed to the same nominal concentrations of PAHs. Gene expression levels were captured after 3 h of PAH exposure by metatranscriptomic analysis. Transcriptomic library sizes are shown in Table S6. Overall, the Mediterranean metatranscriptome showed a higher number of PAH-related transcripts significantly enriched in the PAH-exposed community in comparison to that of the Antarctica, consistent with the PAH biodegradation observed (high in Mediterranean waters, essentially null in Antarctic waters, Fig. 1). In the Mediterranean, SAR11 clade, Rhodobacterales, and other Alphaproteobacteria accumulated the highest number of significant increases in the relative transcript abundances at the PAH-exposed water compared to controls (*t*-test, *p* < 0.05), suggesting that this Alphaproteobacterial community was well adapted to PAH disturbance (Fig. 2). This result contrasts with the general common bloom of HC Gammaproteobacteria following pulses of PAH in oil spills scenarios (Mason et al. 2012; King et al. 2015), but it is in agreement with other observations under low concentrations of ADOC compounds (Martinez-Varela et al. 2021).

**Fig. 3:**
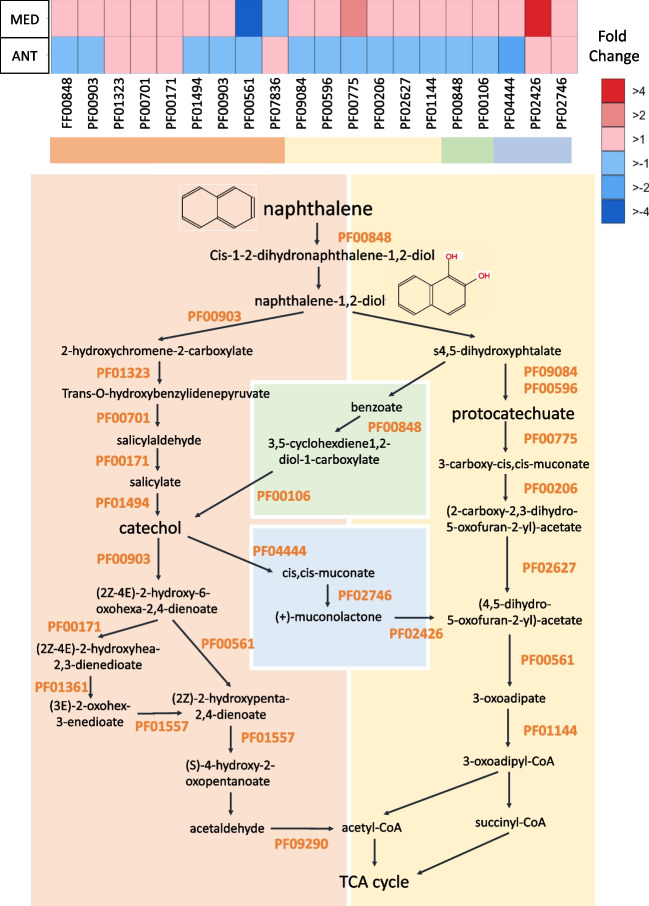
Fold-change in expression of genes involved in PAH degradation between PAH treatments and controls measured by metatranscriptomics. PAH degradation pathway is represented for the naphthalene (model and simplest PAH compound). The list of the specific Pfam profiles involved in PAH degradation is listed in Table S2

Specific search for PAH degradation-related genes showed that the relative abundance of most of them increased at the PAH-exposed Mediterranean community, but reduced at Antarctica PAH-incubated samples, consistent with the measured PAH removal in the experiments (Fig. 3). The ring hydroxylating dioxygenase (RHD), catalyzer complex of the ring-opening PAH dihydroxylation (PF00848), was found significantly enriched at the Mediterranean PAH-exposed community for those transcripts affiliated to Rhodobacterales and to Proteobacteria other than Alpha- and Gammaproteobacteria groups. At the same site, different taxa showed significantly higher transcription levels for transcripts involved in the steps following the ring hydroxylation towards catechol and protocatechuate pathway (Fig. S2). The immediate enrichments of PAH degrading genes in the Mediterranean, combined with the degradation rates observed at this site, suggest this naturally occurring community must have taken part in the biodegradation of less hydrophobic PAH.

To widen the understanding of bacterial HGT functioning under PAH exposure at low concentrations, specific transposon families were searched within the different metatranscriptomes. Transcripts identified as phage integrases and transposon-related genes DDE_Tnp_1, rve, Y1 and Y2, Tnp, and TnpB showed significant enrichments in the PAH-exposed Mediterranean samples (Fig. 2). This result suggests that (1) PAH exposure triggered immediate HGT events, which are known to confer a selective advantage in front of other toxicant exposures at high concentrations such as antibiotics, metals, and other synthetic pollutants (Wright et al. 2008; Lekunberri et al. 2018), and (2) that the naturally occurring microbial population showed a high gene plasticity, harboring the genetic potential for gene transfer (Cerro-Gálvez et al. 2021). In fact, MGE-mediated HGT is considered the main adaptive trait in freshwater and soil ecosystems acutely contaminated by industrial pollutants (Wright et al. 2008; Sobecky and Hazen 2009). MGE trigger the activation of degradation metabolic pathways within the community, either favoring the removal of toxicants in the media or biosurfactant production to facilitate the bioavailability of the compounds (Cerro-Gálvez et al. 2021). This study provides further experimental evidence on the role played by ADOC as an environmental stressor promoting local adaptation of microbial communities to background pollution in seawater. Furthermore, transcripts assigned to membrane transport and stress response showed 1.5- and 2.1-fold increases, respectively, at the PAH-exposed Mediterranean metatranscriptome compared to controls. In particular, these transcripts were significantly enriched for Alphaproteobacteria clades not included in the SAR11 group and Rhodobacterales groups. These results are consistent with previous reports showing that functions related to the activation of stress responses, membrane transportation, and HGT constitute microbial responses to ADOC exposure at environmentally relevant concentrations (Cerro-Gálvez et al. 2019; Martinez-Varela et al. 2021; Cerro-Gálvez et al. 2021).

In the Antarctica, the taxonomic transcripts affiliation remained mostly unchanged after PAH incubation (Fig. 2, Fig. S2, and Fig. S3), which induced fewer changes in relative transcript contribution than the ones observed in the Mediterranean experiments. The only significant changes in transcript abundance related to the exposure to PAHs corresponded to an enrichment of phosphorus metabolism-related transcripts from the Pseudomonadales group, and to a depletion of transcripts related to either nitrogen metabolism affiliated to Rhodobacterales or to cofactors, vitamins, and prosthetic groups and to regulation and cell signalling functions affiliated to Sphingobacteriales. Transcripts related to gene transfer in the Antarctic microbiome remained unaltered after exposure to PAHs (Fig. 2).

### Changes in community composition after PAH exposure

**Fig. 4:**
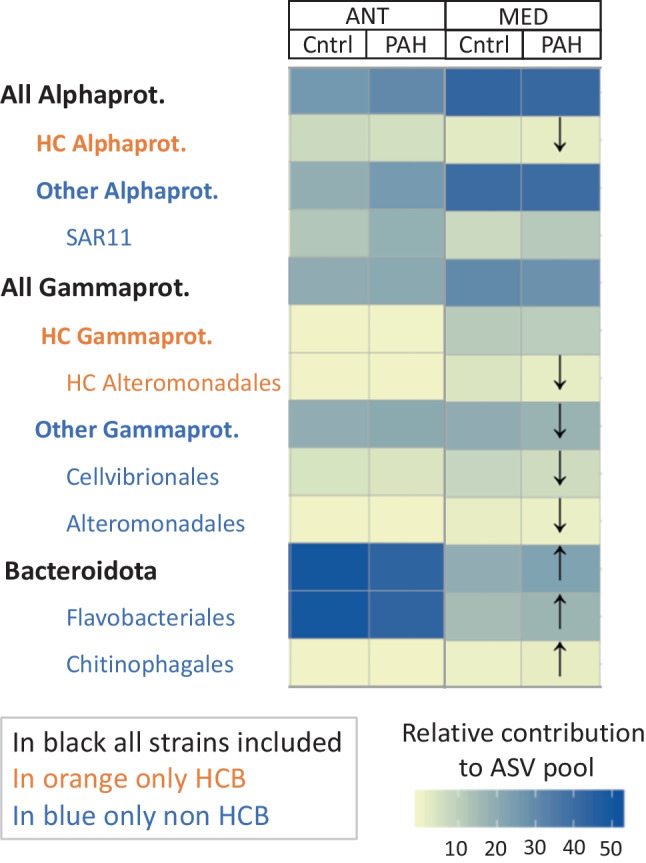
Changes of bacterial composition of the free-living fraction in 48 hours PAH exposure experiments (PAH in the figure) and controls (Cntrl, with no PAH addition). Arrows indicate significant changes in the relative abundances between control and PAH exposed communities based on t-test, *p* < 0.05

Significant changes in community composition between controls and PAH treatments were observed in the Mediterranean microbiomes after 48 h of exposure (PERMANOVA, *p* = 0.03, Fig. 4 and Fig. S4). Non-HC gammaproteobacterial groups significantly decreased their contribution to the 16S pool from the PAH-treated samples, whereas other dominant groups (Bacteroidota and Flavobacteriales) significantly increased their relative abundance (Fig. 4, Table S7). In Antarctica, no statistically significant changes were observed between control and PAH-exposed communities (Fig. 4).

The presence of reported HC bacterial strains was detected in both sites (Table S8). In the Mediterranean, HC Alphaproteobacteria and HC Alteromonadales relative abundances significantly decreased in the PAH-treated samples (Fig. 4, Table S7). In fact, PAH exposed communities showed a general decline (1.8-fold to 4.7-fold ) for different genera of HC bacterial strains, including the HC Actinobacteria genera *Arthrobacter*; HC Gammaproteobacteria genera *Glaciecola, Thalassotalea, Bermanella, Oleiphiluls*, and *Alteromonas*; and HC Alphaproteobacteria genera *Sulfitobacter, Jannaschia*, and *Tropicibacter* (Fig. S4). The opposite occurred in Antarctica, where HCB low abundant taxa belonging to the rare biosphere (relative abundance < 0.03%), clearly increased their relative contribution to the total pool of ASV (Fig. S4). For instance, the copiotroph HC Gammaproteobacteria genera *Pseudoalteromonas* showed a 5.9-fold increase in the PAH-exposed community compared to controls (Fig. S4). Some of these copiotrophic HCB strains, such as HCB belonging to Alteromonadales (*Glaciecola*, *Alteromonas*, *Pseudoalteromonas*, and *Thalassotalea*), are found ubiquitously at low abundances, but exponentially increase their populations in the immediate aftermath of marine oil spills (Kleindienst et al. 2016), becoming key players at the initial stages of the microbial succession of hydrocarbon degradation (Kostka et al. 2011; Dubinsky et al. 2013). These results contrast with other experimental observations in polar and subpolar marine systems, where significant microbial community shifts were observed after short exposure times under ADOC background concentrations (24 h) (Cerro-Gálvez et al. 2019; Martinez-Varela et al. 2022). This could be due to the fact that microbial PAH removal and HCB succession is conditioned by multiple factors such as the concentrations of other available carbon sources in the environment, nutrient availability, or the temperature (Ghosal et al. 2016; Cerro-Gálvez et al. 2019). Also, these contrasting trends between sites suggest that the pace at which microbial communities respond to low concentrations of PAH is site specific. In the Mediterranean, copiotrophic HCB subpopulation is already retreating after 48 h, suggesting a faster microbial response in front of PAH perturbation in comparison to Antarctica’s microbiome.

## Conclusion

Overall, our results show that the Mediterranean microbiome responded faster than that of the Antarctica to the PAH exposure at environmentally relevant concentration. In the Mediterranean, we observed the immediate activation of transcripts related to stress response mechanisms in front of PAH toxicity, horizontal gene-transfer events, and PAH metabolism. Following this immediate response, significant changes in the community composition after 48 h from the initial PAH exposure were also observed. At the Mediterranean site, the Alphaproteobacterial community seemed to be well adapted to ADOC perturbation, showing the highest number of transcript significant enrichments after 3 h. Aligned to these compositional and functional responses, the concentrations of the less hydrophobic LMW PAH were significantly reduced after 48 h in the Mediterranean, indicating that these compounds are readily biodegraded by this natural community. This observation can explain the fact that even though the Mediterranean receives important PAH inputs from proximate regions, the PAH concentrations in seawater are relatively low.

### Supplementary information

ESM 1

## Data Availability

The datasets analyzed during the current study are available from European Nucleotide Archive (ENA) at EMBL-EBI under accession number PRJEB55141.
