# Supplementary material for: Metatranscriptomic responses and microbial degradation of background polycyclic aromatic hydrocarbons in the coastal Mediterranean and Antarctica
Source: Environ Sci Pollut Res Int. 2023 Nov 7;30(57):119988–99. doi: 10.1007/s11356-023-30650-1 (PMC10697874; doi:10.1007/s11356-023-30650-1)
Supplement: Supplementary file 1 — ESM 1 [file 11356_2023_30650_MOESM1_ESM.docx]

**Supplemental Material**

**Metatranscriptomic responses and microbial degradation of polycyclic aromatic hydrocarbons in the coastal Mediterranean and Antarctica**

**Alícia Martinez-Varela^1^, Gemma Casas^1^, Naiara Berrojalbiz^1^, Daniel Lundin^2^, Jordi Dachs^1^, Benjamin Piña^1^, and Maria Vila-Costa^1*^.**

^1^ Department of Environmental Chemistry, Institute of Environmental Assessment and Water Research, IDAEA-CSIC; Barcelona, Catalunya, Spain

^2^ ﻿﻿Centre for Ecology and Evolution in Microbial Model Systems, EEMiS, Linnaeus University, Kalmar 35195, Sweden

*Corresponding author: Maria Vila-Costa

Email: [maria.vila@idaea.csic.es](mailto:maria.vila@idaea.csic.es)

OrcID:  [**000-0003-1730-8418**](http://orcid.org/0000-0003-1730-8418)

**Table S1.** PAH dissolved and particulate phase concentrations in each experimental site and treatment at initial time point (ng L^-1^) (Part: Particulate phase concentrations, Diss: Dissolved phase concentrations, FL: Fluorene, ANT: Anthracene, PHE: Phenantrene, PYR: Pyrene, FLT: Fluoranthene, CRY: Chrysene, BaA: Benz(a)anthracene, BbF : Benzo(b)fluoranthene, BkF: Benzo(k)fluoranthene, BaP: Benzo(a)pyrene; DBA: Dibenz(a,h)anthracene, IPY : Indeno(1,2,3-cd)pyrene, BghP: Benzo(ghi)perylene)

|  |  | Control | | | PAH | | |
| --- | --- | --- | --- | --- | --- | --- | --- |
|  |  | Diss. | Part. | Total | Diss. | Part. | Total |
| Antarctica | FL | 2.38 ± 1.44 | 0.61 ± 0.15 | 2.99 ± 1.59 | 11.44 ± 3.43 | 0.06 ± 0.02 | 11.50 ± 3.45 |
|  | ANT | 0.26 ± 0.05 | 0.37 ± 0.03 | 0.63 ± 0.08 | 47.27 ± 19.29 | 0.47 ± 0.19 | 47.74 ± 19.48 |
|  | PHE | 2.21 ± 1.06 | 1.04 ± 0.07 | 3.25 ± 1.14 | 19.33 ± 3.90 | 0.20 ± 0.04 | 19.53 ± 3.94 |
|  | PYR | 1.36 ± 0.35 | 1.83 ± 0.16 | 3.20 ± 0.50 | 163.82 ± 7.88 | 4.33 ± 0.21 | 168.15 ± 8.09 |
|  | FLT | 1.51 ± 0.54 | 1.57 ± 0.13 | 3.08 ± 0.67 | 194.75 ± 6.68 | 9.75 ± 0.33 | 204.50 ± 7.01 |
|  | CRY | 1.68 ± 0.41 | 1.17 ± 0.02 | 2.85 ± 0.43 | 12.08 ± 2.95 | 2.21 ± 0.54 | 14.29 ± 3.49 |
|  | BaA | 1.75 ± 0.94 | 0.51 ± 0.12 | 2.27 ± 1.06 | 44.54 ± 9.11 | 8.74 ± 1.79 | 53.28 ± 10.89 |
|  | BbF | 0.29 ± 0.29 | 0.23 ± 0.09 | 0.52 ± 0.38 | 10.46 ± 2.57 | 2.15 ± 0.53 | 12.61 ± 3.09 |
|  | BkF | 0.38 ± 0.16 | 0.29 ± 0.04 | 0.67 ± 0.20 | 3.07 ± 0.91 | 1.33 ± 0.40 | 4.40 ± 1.31 |
|  | BaP | 0.00 ± 0.00 | 0.13 ± 0.03 | 0.13 ± 0.03 | 9.07 ± 2.12 | 4.13 ± 0.96 | 13.20 ± 3.08 |
|  | DBA | 0.19 ± 0.19 | 0.24 ± 0.11 | 0.42 ± 0.29 | 2.95 ± 1.43 | 3.12 ± 1.52 | 6.07 ± 2.95 |
|  | IPY | 1.31 ± 0.30 | 1.19 ± 0.06 | 2.51 ± 0.36 | 5.45 ± 2.21 | 6.92 ± 2.81 | 12.38 ± 5.02 |
|  | BghP | 0.64 ± 0.24 | 0.82 ± 0.10 | 1.46 ± 0.33 | 2.90 ± 0.99 | 4.13 ± 1.41 | 7.03 ± 2.41 |
|  | **TOTAL** | **13.97 ± 5.96** | **10.01 ± 1.11** | **23.98 ± 7.06** | **527.14 ± 63.47** | **47.55 ± 10.74** | **574.69 ± 74.21** |
| Mediterranean | FL | 1.03 ± 0.08 | 0.10 ± 0.01 | 1.13 ± 0.09 | 198.85 ± 94.93 | 0.83 ± 0.40 | 199.68 ± 95.33 |
|  | ANT | 0.17 ± 0.03 | 0.01 ± 0.00 | 0.18 ± 0.03 | 104.54 ± 7.36 | 0.74 ± 0.05 | 105.28 ± 7.41 |
|  | PHE | 3.10 ± 0.45 | 0.08 ± 0.02 | 3.18 ± 0.46 | 94.19 ± 2.58 | 0.68 ± 0.02 | 94.86 ± 2.60 |
|  | PYR | 0.88 ± 0.08 | 0.04 ± 0.01 | 0.92 ± 0.09 | 269.89 ± 1.05 | 4.35 ± 0.02 | 274.25 ± 1.07 |
|  | FLT | 0.74 ± 0.08 | 0.02 ± 0.00 | 0.76 ± 0.08 | 341.53 ± 0.73 | 9.45 ± 0.02 | 350.98 ± 0.75 |
|  | CRY | 0.09 ± 0.00 | 0.01 ± 0.00 | 0.10 ± 0.01 | 57.86 ± 9.94 | 4.80 ± 0.82 | 62.66 ± 10.76 |
|  | BaA | 0.29 ± 0.00 | 0.00 ± 0.00 | 0.29 ± 0.00 | 145.52 ± 16.80 | 12.80 ± 1.48 | 158.31 ± 18.28 |
|  | BbF | 0.00 ± 0.00 | 0.00 ± 0.00 | 0.00 ± 0.00 | 38.07 ± 4.50 | 3.48 ± 0.41 | 41.55 ± 4.91 |
|  | BkF | 0.00 ± 0.00 | 0.00 ± 0.00 | 0.00 ± 0.00 | 15.74 ± 2.99 | 2.72 ± 0.52 | 18.46 ± 3.50 |
|  | BaP | 0.00 ± 0.00 | 0.00 ± 0.00 | 0.00 ± 0.00 | 35.69 ± 4.25 | 6.40 ± 0.76 | 42.09 ± 5.01 |
|  | DBA | 0.00 ± 0.00 | 0.00 ± 0.00 | 0.00 ± 0.00 | 9.29 ± 1.05 | 3.40 ± 0.39 | 12.70 ± 1.44 |
|  | IPY | 0.02 ± 0.00 | 0.01 ± 0.00 | 0.03 ± 0.00 | 25.22 ± 4.28 | 10.77 ± 1.83 | 36.00 ± 6.11 |
|  | BghP | 0.00 ± 0.00 | 0.02 ± 0.00 | 0.02 ± 0.00 | 8.29 ± 0.89 | 3.90 ± 0.42 | 12.20 ± 1.30 |
|  | **TOTAL** | **6.31 ± 0.72** | **0.30 ± 0.04** | **6.61 ± 0.76** | **1344.69 ± 45.78** | **64.32 ± 6.26** | **1409.01 ± 39.52** |

**Table S2**. Pfam profiles of proteins involved in PAH degradation and horizontal gene tranasfeer. (Pfam: The protein families database number)

| Pfam | Function | Name |
| --- | --- | --- |
| PF00848 | Upstream PAH degradation  pathway | Ring-hydroxylating dioxygenase |
| PF00171 |  | Salicylaldehyde dehydrogenase |
| PF00596 |  | 3,4-dihydroxyphthalate decarboxylase |
| PF00701 |  | Dihydrodipicolinate synthetase |
| PF00775 |  | Protocatechuate 3,4-dioxygenase beta chain |
| PF00106 |  | 1,6-dihydroxycyclohexa-2,4-diene-1-carboxylate dehydrogenase |
| PF00903 |  | Glyoxalase dioxygenase |
| PF01323 |  | 2-hydroxychromene-2-carboxylate isomerase |
| PF01494 |  | Salicylate hydroxylase |
| PF09084 |  | 4,5-dihydroxyphthalate decarboxylase |
| PF00561 | Catechol degradation pathway | 2-hydroxymuconate semialdehyde hydrolase |
| PF00903 |  | Metapyrocatechase |
| PF02746 |  | Muconate cycloisomerase 1 |
| PF04444 |  | Catechol 1,2-dioxygenase |
| PF07836 |  | 4-hydroxy-2-oxovalerate aldolase |
| PF02426 |  | Muconolactone Delta-isomerase |
| PF00206 | Benzoate and protocatechuate degradation pathway | 3-carboxy-cis,cis-muconate cycloisomerase |
| PF00561 |  | 3-oxoadipate enol-lactonase |
| PF01144 |  | Coenzyme A transferase |
| PF02627 |  | 4-carboxymuconolactone decarboxylase |
| PF00665 | DNA transposons | Retroviral integrase (rve) |
| PF01609 |  | Transposase 11  superfamily (DDE_Tnp_1) |
| PF00589 |  | Phage integrase |
| PF01797 |  | Transposase 17 (Y1_Tnp) |
| PF05717 |  | Transposase 34 (TnpB_IS66) |
| PF04986 |  | Transposase 32 (Y2_Tnp) |

**Table S3.** Biotic and abiotic features at initial conditions and over the course of the incubation for each experimental condition.

(* indicate significant differences between treatment and control in that time-point, paired t-test, p<0.05) . BA: bacterial abundance, HNA and LNA: high- and low- nucleic acid content heterotrophic bacterial cells

|  | **ANTARCTICA** | | | | | **MEDITERRANEAN** | | | | |
| --- | --- | --- | --- | --- | --- | --- | --- | --- | --- | --- |
|  | **Initial conditions** | **T3** | | **T48** | | **Initial conditions** | **T3** | | **T48** | |
|  |  | **Control** | **PAH** | **Control** | **PAH** |  | **Control** | **PAH** | **Control** | **PAH** |
| **Latitude** | 62.63 S | | | | | 41.67 N | | | | |
| **Longitude** | 60.4 W | | | | | 2.8 E | | | | |
| **Sea Surface Temperature (ºC)** | 1.46 |  |  |  |  | 26.40 |  |  |  |  |
| **Salinity (PSU)** | 33.49 |  |  |  |  | 38.02 |  |  |  |  |
| **All BA**  **(Cells mL^-1^)** | 338000 ± 95767.08 | 244750 ± 19050.37 | 181777.78 ± 111423.91 | 501750 ± 78321.23 | 498125 ± 97775.89 | 602250± 18661.46 | 638750 ± 8770.21 | 698625 ± 35387.40 | 805000 ± 38444.77 | 743500 ± 89061.78 |
| **HNA**  **(Cells mL^-1^)** | 243750 ± 73031.39 | 175750 ± 16839.93 | 111246.67 ± 97496.28 | 379875 ± 68861.01 | 364875 ± 74060.67 | 249750± 9810.71 | 328000 ± 4760.95 | 302500 ± 54542.25 | 545750 ± 16028.62 | 482625 ± 83718.81 |
| **LNA**  **(Cells mL^-1^)** | 94175 ± 25447.77 | 69450 ± 8306.82 | 70655.56 ± 20918.30 | 121875 ± 12040.85 | 132812.50 ± 47071.33 | 352500± 10279.43 | 310750 ± 6652.07 | 396250 ± 33648.39 | 259000 ± 26267.85 | 261000 ± 34570.84 |
| **N-NH_4_^+^**  **(μmol L^-1^)** | 7.43 ± 1.18 | 2.73 ± 2.47 | 2.40 ± 1.90 | 6.07 ± 3.08 | 8.02 ± 4.05 | n.d. | 0.17 ± 0.16 | 0.11 ± 0.02 | 2 ± 2.10 | 0.20 ± 0.20 |
| **NO^−3^ + NO^-2^**  **(μmol L^-1^)** | 14.06 ± 0.72 | 23.93 ± 2.59 | 25.01 ± 1.46 | 24.75 ± 0.12 | 20.08 ± 4.43 | n.d. | 2.18 ± 0.01 | 0.39 ± 0.12 (*) | 3.17 ± 0.08 | 0.82 ± 0.25 (*) |
| **PO_4_^3-^**  **(μmol L^-1^)** | 1.11 ± 0.03 | 1.60 ± 0.14 | 1.68 ± 0.07 | 1.65 ± 0.02 | 1.45 ± 0.21 | n.d. | 0.12 ± 0.12 | 0.04 ± 0.01 | 0.02 ± 0 | 0.02 ± 0 |
| **Shannon diversity Index 16S ASV** | 1.20 ± 0.21 | 1.56 ± 0.30 | 2.30 ± 0.77 | 3.40 ± 0.01 | 3.44 ± 0.04 | 4.58 | 4.61 ± 0.10 | 4.66 ± 0.08 | 4.66 ± 0.01 | 4.77 ± 0.08 |

**Table S4.** Community composition of microbial communities at initial time point (T3) measured by 16S rRNA gene amplicon sequencing (Relative abundances in % of total reads and variability expressed in standard deviation, HC : Hydrocarbonoclastic). Notice that counts of general taxonomical groups include HC bacteria.

|  | Antarctica | | Mediterranean | |
| --- | --- | --- | --- | --- |
|  | Control | PAH | Control | PAH |
| Alphaproteobacteria | 16.35 ± 1.70 | 17.67 ± 12.46 | 45.74 ± 0.85 | 45.32 ± 0.44 |
| SAR11 clade | 9.20 ± 4.26 | 11.76 ± 12.38 | 7.67 ± 0.95 | 11.80 ± 2.25 |
| Rhodobacterales | 4.67 ± 3.82 | 1.96 ± 0.99 | 3.75 ± 0.02 | 3.01 ± 0.08 |
| Rickettsiales | 1.67 ± 0.94 | 3.01 ± 0.84 | 18.22 ± 0.44 | 14.38 ± 1.22 |
| Gammaproteobacteria | 28.37 ± 0.37 | 25.70 ± 8.16 | 16.61 ± 0.04 | 19.20 ± 0.21 |
| Alteromonadales | 6.00 ± 3.99 | 3.21 ± 2.49 | 0.31 ± 0.07 | 0.72 ± 0.05 |
| Cellvibrionales | 4.65 ± 1.51 | 5.32 ± 1.36 | 6.37 ± 0.01 | 6.65 ± 0.56 |
| Oceanospirillales | 11.70 ± 3.08 | 11.84 ± 5.44 | 1.52 ± 0.20 | 1.66 ± 0.04 |
| Pseudomonadales | 0.31 ± 0.34 | 0.17 ± 0.09 | 0.05 ± 0.04 | 0.03 |
| Thiotrichales | 1.71 ± 0.07 | 1.49 ± 0.86 | 0 | 0 |
| Bacteroidota | 53.87 ± 0.48 | 54.54 ± 5.98 | 27.00 ± 0.27 | 26.45 ± 1.63 |
| Sphingobacteriales | 51.77 ± 0.59 | 52.95 ± 6.89 | 20.02 ± 0.47 | 19.54 ± 1.52 |
| Flavobacteriales | 0.09 ± 0.03 | 0.07 ± 0.06 | 0.63 ± 0.05 | 0.64 ± 0.02 |
| Cyanobacteria | 0 | 0 | 7.05 ± 0.97 | 5.90 ± 1.23 |
| Other Bacterial groups | 0.44 ± 0.26 | 0.42 ± 0.28 | 0.28 ± 0.08 | 0.22 ± 0.01 |
|  |  |  |  |  |
| HC Actinobacteria | 0.07 | 0.29 ± 0.30 | 0.17 ± 0.03 | 0.07 ± 0.01 |
| *Arthrobacter* | 0 | 0.18 | 0.17 ± 0.03 | 0.06 ± 0.00 |
| *Nocardioides* | 0.07 | 0.20 ± 0.17 | 0 | 0.02 |
| HC Alphaproteobacteria | 4.20 ± 3.24 | 7.30 ± 9.06 | 0.16 ± 0.00 | 0.46 ± 0.07 |
| *Jannaschia* | 0 | 0 | 0.15 ± 0.02 | 0.42 ± 0.09 |
| HC Gammaproteobacteria | 6.03 ± 4.25 | 3.23 ± 2.54 | 0.83 ± 0.21 | 2.49 ± 0.83 |
| HC Alteromonadales | 5.42 ± 3.79 | 2.86 ± 2.30 | 0.08 ± 0.04 | 0.30 ± 0.02 |
| *Colwellia* | 4.73 ± 3.19 | 2.51 ± 2.15 | 0 | 0 |
| *Glaciecola* | 0 | 0 | 0.03 | 0.07 ± 0.01 |
| *Thalassotalea* | 0.14 | 0.23 | 0.01 | 0.03 |
| *Pseudoalteromonas* | 0.62 ± 0.69 | 0.28 ± 0.18 | 0.06 ± 0.03 | 0.21 ± 0.00 |
| HC Pseudomonadales | 0.31 ± 0.34 | 0.17 ± 0.09 | 0.05 ± 0.04 | 0.03 |
| *Pseudomonas* | 0 | 0.08 ± 0.05 | 0 | 0 |
| HC Vibrionales | 0 | 0 | 0.68 ± 0.21 | 2.14 ± 0.88 |
| HC Bacteroidia | 0.24 ± 0.21 | 0.12 ± 0.12 | 0.04 ± 0.02 | 0 |
| All HCB | 10.50 ± 1.17 | 10.87 ± 7.60 | 1.21 ± 0.26 | 3.02 ± 0.88 |

**Table S5.** Some reported PAH levels in the Mediterranean Sea and the maritime Antarctica

| **Region** | **PAH compounds** | **Water body** | **Concentration range** | | **Reference** |
| --- | --- | --- | --- | --- | --- |
| **Mediterranean** | | | | | |
| Central Mediterranean (Sea Sarno river outlet Gulf of Naples,Tyrrhenian Sea) | The Σ_16_ PAHs identified by the USEPA as priority pollutants and perylene | Surface sea water | 23.1 to 2670.4 ng L^−1^ in water | | Montuori & Triassi, 2012 |
| Southern Eastern Mediterranean Sea (Alexandria’s coastal water, Egypt) | Σ_16_ PAHs identified by the USEPA as priority pollutants | Surface sea water | 13.4 and 6076 ng L^−1^ | | Elnaggar et al., 2018 |
| Eastern Mediterranean | Σ_19_ PAH | Surface sea water | 161 to 8,797 pg L^−1^ (dissolved) and  33 to 319 pg L^−1^ (particulate) | | Berrojalbiz et la., 2011 |
| Western Mediterranean | Σ_19_ PAH | Surface sea water | 158 to 808 pg L^−1^ (dissolved) and 33 to 369 pg L^−1^ (particulate) | | Berrojalbiz et la., 2011 |
| NW Mediterranean Sea (French coast) | Σ_17_ PAH | Surface sea water | 4.7 · 10^3^ -1.5· 10^5^ pg L^−1^ | | Guigue et al., 2011 |
| **Antarctica** | | | | | |
| Terra Nova Bay, Antarctica | Σ_13_ PAHs | Surface sea water | 2.24 to 4.01 ng L^−1^ (disolved)  m 1.65 to 3.65 ng L^−1^ (particulate) | Cincinelli et al., 2005 | |
| Gerlache Inlet sea, Antarctica | Σ_13_ PAHs | Surface sea water | ﻿5.27–9.43 ng L^−1^ | Stortini et al., 2009 | |
| Gerlache Inlet sea, Antarctica | Σ_14_ PAHs | Surface sea water | ﻿331 pg L^−1^ (dissolved) | Fuoco et al., 2005 | |
| South Shetland Islands, Antarctica | Σ_25_ PAHs | Suspended particulate matter | 30–82 ng g-1 dw | Curtosi et al., 2009 | |
| Coastal Livingston island,  South Shetland Islands, Antarctica | Σ_13_ PAHs | Surface sea water | 1.0 ± 0.82 ng L −1 | Casal et al., 2018 | |

**Table S6.** Metatranscriptomic data library sizes (after lowly expressed genes removal at CPM < 0.5, which corresponds to a count of 10-15 for the library sizes)

| **Site** | **sample** | **Library size** | **treatment** | **replicate** | **filter_fraction** |
| --- | --- | --- | --- | --- | --- |
| Antarctica | AE4599 | 11088452 | Control | A | 0.2 |
| Antarctica | AE4600 | 8749016 | Control | B | 0.2 |
| Antarctica | AE4604 | 10620420 | PAH | A | 0.2 |
| Antarctica | AE4605 | 9780415 | PAH | B | 0.2 |
| Antarctica | AE4606 | 12638738 | PAH | C | 0.2 |
| Antarctica | AE4608 | 10922491 | Control | A | 3 |
| Antarctica | AH2690 | 9452798 | Control | B | 3 |
| Antarctica | AE4612 | 12827161 | PAH | A | 3 |
| Antarctica | AE4613 | 12346906 | PAH | B | 3 |
| Antarctica | AE4614 | 13932804 | PAH | C | 3 |
| Mediterranean | S4_34UD | 25230565 | Control | A | 0.2 |
| Mediterranean | S5_142UDI | 20310295 | Control | B | 0.2 |
| Mediterranean | S5_118UDI | 15332951 | PAH | A | 0.2 |
| Mediterranean | S5_130UDI | 17286971 | PAH | B | 0.2 |

**Table S7.** Community composition of microbial communities at final time point (T48) measured by 16S rRNA gene amplicon sequencing (Relative abundances in % of total reads and variability expressed in standard deviation, HC : Hydrocarbonoclastic). Notice that counts of general taxonomical groups include HC bacteria.

|  | Antarctica | | Mediterranean | |
| --- | --- | --- | --- | --- |
|  | Control | PAH | Control | PAH |
| Alphaproteobacteria | 26.96 ± 2.74 | 32.23 ± 0.41 | 44.50 ± 0.63 | 43.22 ± 2.47 |
| SAR11 clade | 12.97 ± 4.09 | 19.61 ± 2.70 | 7.82 ± 0.67 | 11.61 ± 1.07 |
| Rhodobacterales | 12.53 ± 7.05 | 11.20 ± 2.62 | 21.14 ± 1.72 | 16.50 ± 1.23 |
| Rickettsiales | 0.58 ± 0.08 | 0.31 ± 0.08 | 2.39 ± 0.41 | 2.49 ± 0.21 |
| Gammaproteobacteria | 20.55 ± 1.09 | 21.41 ± 1.78 | 31.55 ± 0.92 | 28.64 ± 2.27 |
| Alteromonadales | 0.08 ± 0.06 | 0.08 ± 0.04 | 6.44 ± 1.31 | 3.16 ± 0.46 |
| Cellvibrionales | 4.75 ± 0.92 | 4.45 ± 0.41 | 8.69 ± 0.33 | 6.71 ± 0.70 |
| Oceanospirillales | 8.59 ± 0.61 | 8.95 ± 1.29 | 4.12 ± 0.34 | 3.97 ± 0.68 |
| Pseudomonadales | 0.03 ± 0.02 | 0.10 ± 0.04 | 0 | 0 |
| Thiotrichales | 0.54 ± 0.05 | 0.59 ± 0.03 | 0 | 0 |
| Bacteroidota | 52.21 ± 3.74 | 45.98 ± 2.16 | 19.72 ± 0.41 | 23.28 ± 0.11 |
| Sphingobacteriales | 0 | 0.01 ± 0.00 | 0.12 ± 0.04 | 0.41 ± 0.09 |
| Flavobacteriales | 51.84 ± 3.75 | 45.63 ± 2.15 | 15.44 ± 0.67 | 17.35 ± 0.11 |
| Cyanobacteria | 0 | 0 | 1.63 ± 0.15 | 2.19 ± 0.15 |
| Other Bacterial groups | 0.03 ± 0.03 | 0.06 ± 0.02 | 0.15 ± 0.01 | 0.42 ± 0.06 |
|  |  |  |  |  |
| HC Actinobacteria | 0.05 | 0.01 ± 0.00 | 0.22 ± 0.11 | 0.04 ± 0.01 |
| *Arthrobacter* | 0.05 | 0.02 | 0.22 ± 0.11 | 0.03 |
| HC Alphaproteobacteria | 7.17 ± 6.14 | 5.89 ± 2.74 | 2.85 ± 0.25 | 2.14 ± 0.24 |
| *Jannaschia* | 0 | 0 | 2.68 ± 0.16 | 2.05 ± 0.26 |
| HC Gammaproteobacteria | 0.09 ± 0.04 | 0.15 ± 0.05 | 11.32 ± 0.73 | 10.67 ± 2.28 |
| HC Alteromonadales | 0.06 ± 0.03 | 0.05 ± 0.05 | 4.41 ± 0.98 | 2.13 ± 0.26 |
| *Colwellia* | 0.05 ± 0.01 | 0.04 ± 0.03 | 0 | 0 |
| *Glaciecola* | 0 | 0.01 | 1.50 ± 0.52 | 0.53 ± 0.22 |
| *Thalassotalea* | 0 | 0.01 | 0.45 ± 0.17 | 0.13 ± 0.04 |
| *Pseudoalteromonas* | 0.02 | 0.04 | 1.28 ± 0.07 | 0.89 ± 0.12 |
| HC Pseudomonadales | 0.03 ± 0.02 | 0.10 ± 0.04 | 0 | 0 |
| *Pseudomonas* | 0 | 0.04 ± 0.03 | 0 | 0 |
| HC Vibrionales | 0 | 0 | 6.16 ± 0.56 | 7.51 ± 1.77 |
| HC Bacteroidia | 0.03 ± 0.02 | 0.03 ± 0.03 | 0 | 0 |
| All HCB | 7.31 ± 6.13 | 6.06 ± 2.71 | 14.39 ± 1.09 | 12.85 ± 2.13 |

**Table S8**. Subset of genera including previously reported hydrocarbonoclastic bacterial strains or strains found in oil polluted marine environments (Takahashi *et al.*, 2013; Lozada *et al.*, 2014; Karthikeyan *et al.*, 2020)detected in the experimental dataset of both sites. Grey boxes indicate presence of that strain in that site. (HC : Hydrocarbonoclastic, Ant: Antarctica, Med: Mediterranean)

| Label | Genera | Ant | Med |
| --- | --- | --- | --- |
| HC Actinobacteria | *Arthrobacter* |  |  |
|  | *Nocardioides* |  |  |
| HC Alphaproteobacteria | *Jannaschia* |  |  |
|  | *Roseovarius* |  |  |
|  | *Sphingomonas * |  |  |
|  | *Sulfitobacter* |  |  |
|  | *Tropicibacter* |  |  |
| HC Bacilli | *Bacillus* |  |  |
| HC Bacteroidia | *Flavobacterium* |  |  |
|  | *Nonlabens* |  |  |
|  | *Paludibacter* |  |  |
| HC Bdellovibrionia | *Bdellovibrio* |  |  |
|  | *Halobacteriovorax* |  |  |
| HC Fusobacteriia | *Psychrilyobacter* |  |  |
| HC Gammaproteobacteria | *Acinetobacter* |  |  |
|  | *Alkanindiges* |  |  |
|  | *Alteromonas* |  |  |
|  | *Bermanella* |  |  |
|  | *Colwellia* |  |  |
|  | *Delftia* |  |  |
|  | *Glaciecola* |  |  |
|  | *Kangiella* |  |  |
|  | *Marinobacter* |  |  |
|  | *Marinomonas* |  |  |
|  | *Neptuniibacter* |  |  |
|  | *Neptunomonas* |  |  |
|  | *Oleibacter* |  |  |
|  | *Oleiphilus* |  |  |
|  | *Oleispira* |  |  |
|  | *Pseudoalteromonas* |  |  |
|  | *Pseudomonas* |  |  |
|  | *Psychrobacter* |  |  |
|  | *Shewanella* |  |  |
|  | *Thalassolituus* |  |  |
|  | *Thalassotalea* |  |  |
|  | *Vibrio* |  |  |
|  | *Woeseia* |  |  |

**Figure S1.** Taxonomical composition of free living bacteria for each experimental site and sampling time point for PAH exposure and control samples.

**Figure S2.** Metatranscripts taxonomical assignation 3 h after PAH exposure. (*PERMANOVA shows no differences in ORFs between treatment and control)


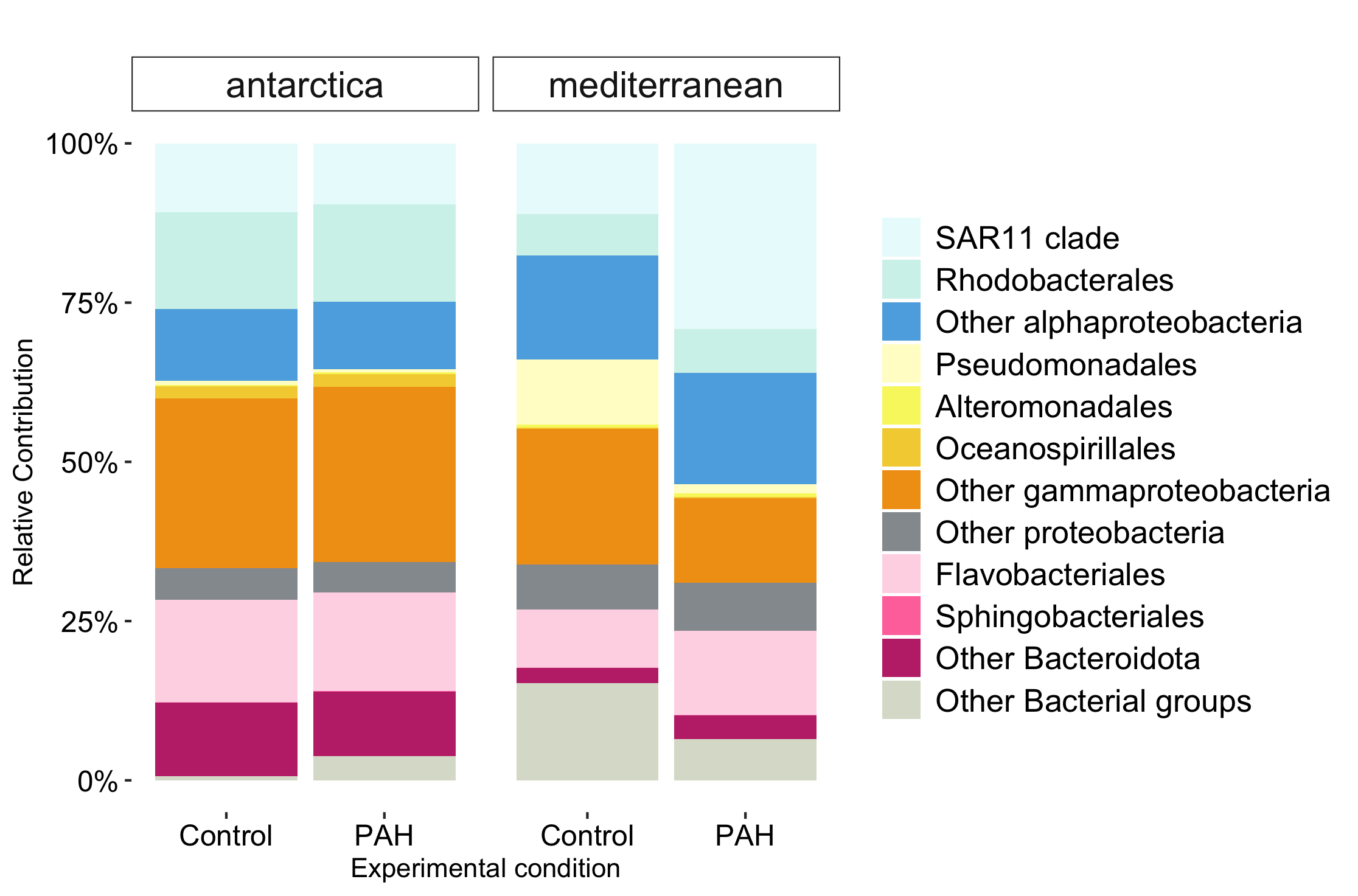


**Figure S3.** Metatrascriptomes taxonomic affiliation fold changes between PAH and control treatments after 3 h of PAH exposure. (HCB includes only HCB strains, nonHCB exclude all HCB strains within that group) (no taxonomic significant differences in relative transcript contribution between control and treatments)

**Figure S4.** Fold change 16S HCB at time 48 between control and PAH exposed community.

**References of Supplemental Material**

Casal, P., Cabrerizo, A., Vila-Costa, M., Pizarro, M., Jiménez, B., & Dachs, J. (2018). Pivotal role of snow deposition and melting driving fluxes of polycyclic aromatic hydrocarbons at Coastal Livingston Island (Antarctica). *Environmental science & technology*, *52*(21), 12327-12337.

Montuori, P., & Triassi, M. (2012). Polycyclic aromatic hydrocarbons loads into the Mediterranean Sea: Estimate of Sarno River inputs. *Marine pollution bulletin*, *64*(3), 512-520.

El-Naggar, N. A., Emara, H. I., Moawad, M. N., Soliman, Y. A., & El-Sayed, A. A. (2018). Detection of polycyclic aromatic hydrocarbons along Alexandria’s coastal water, Egyptian Mediterranean Sea. *The Egyptian Journal of Aquatic Research*, *44*(1), 9-14.

Berrojalbiz, N., Dachs, J., Ojeda, M. J., Valle, M. C., Castro‐Jiménez, J., Wollgast, J., ... & Zaldivar, J. M. (2011). Biogeochemical and physical controls on concentrations of polycyclic aromatic hydrocarbons in water and plankton of the Mediterranean and Black Seas. *Global Biogeochemical Cycles*, *25*(4).

Guigue, C., Tedetti, M., Giorgi, S., & Goutx, M. (2011). Occurrence and distribution of hydrocarbons in the surface microlayer and subsurface water from the urban coastal marine area off Marseilles, Northwestern Mediterranean Sea. *Marine pollution bulletin*, *62*(12), 2741-2752.

Cincinelli, A., Stortini, A. M., Checchini, L., Martellini, T., Del Bubba, M., & Lepri, L. (2005). Enrichment of organic pollutants in the sea surface microlayer (SML) at Terra Nova Bay, Antarctica: influence of SML on superficial snow composition. *Journal of Environmental Monitoring*, *7*(12), 1305-1312.

Stortini, A. M., Martellini, T., Del Bubba, M., Lepri, L., Capodaglio, G., & Cincinelli, A. (2009). n-Alkanes, PAHs and surfactants in the sea surface microlayer and sea water samples of the Gerlache Inlet sea (Antarctica). *Microchemical Journal*, *92*(1), 37-43.

Fuoco, R., Giannarelli, S., Wei, Y., Abete, C., Francesconi, S., & Termine, M. (2005). Polychlorobiphenyls and polycyclic aromatic hydrocarbons in the sea-surface micro-layer and the water column at Gerlache Inlet, Antarctica. *Journal of Environmental Monitoring*, *7*(12), 1313-1319.

Curtosi, A., Pelletier, E., Vodopivez, C. L., & Mac Cormack, W. P. (2009). Distribution of PAHs in the water column, sediments and biota of Potter Cove, South Shetland Islands, Antarctica. *Antarctic Science*, *21*(4), 329-339.
